# Quantitative proteomics reveals oxygen-induced adaptations in *Caldalkalibacillus thermarum* TA2.A1 microaerobic chemostat cultures

**DOI:** 10.3389/fmicb.2024.1468929

**Published:** 2024-10-28

**Authors:** Samuel I. de Jong, Martijn Wissink, Kadir Yildirim, Martin Pabst, Mark C. M. van Loosdrecht, Duncan G. G. McMillan

**Affiliations:** ^1^Department of Biotechnology, Delft University of Technology, Delft, Netherlands; ^2^School of Biological Sciences, University of Reading, Whiteknights, United Kingdom

**Keywords:** chemostat, regulation, adaptation, alkaliphile, microaerobic, respiration

## Abstract

The thermoalkaliphile *Caldalkalibacillus thermarum* possesses a highly branched respiratory chain. These primarily facilitate growth at a wide range of dissolved oxygen levels. The aim of this study was to investigate the regulation of *C. thermarum* respiratory chain. *C. thermarum* was cultivated in chemostat bioreactors with a range of oxygen levels (0.25% O_2_–4.2% O_2_). Proteomic analysis unexpectedly showed that both the type I and the type II NADH dehydrogenase present are constitutive. The two terminal oxidases detected were the cytochrome *c*:oxygen *aa*_3_ oxidase, whose abundance was highest at 4.2% O_2_. The cytochrome *c*:oxygen *ba*_3_ oxidase was more abundant at most other O_2_ levels, but its abundance started to decline below 0.42% O_2_. We expected this would result in the emergence of the cytochrome *c*:oxygen *bb*_3_ complex or the menaquinol:oxygen *bd* complex, the other two terminal oxidases of *C. thermarum*; but neither was detected. Furthermore, the sodium-proton antiporter complex Mrp was downregulated under the lower oxygen levels. Normally, in alkaliphiles, this enzyme is considered crucial for sodium homeostasis. We propose that the existence of a sodium:acetate exporter decreases the requirement for Mrp under strong oxygen limitation.

## Introduction

Branched respiratory chains are a common feature in the microbial world. Equipped with alternatives for canonical electron transfer chain (ETC) complexes, microbes can effectively manage fluctuating oxygen conditions encountered within their native environments (Poole and Cook, 2000). As an example, the gut microbe *Escherichia coli* has a terminal *bo*_3_ oxidase for aerobic conditions and two *bd*-type terminal oxidases for anaerobic conditions (Musser et al., 1993; Lindqvist et al., 2000); the latter condition would be encountered more often by *E. coli* (Kaper et al., 2004). While prior research under controlled conditions predominantly focused on model organisms (De Graef et al., 1999; Alexeeva et al., 2002; de Groot et al., 2007; Steinsiek et al., 2011, 2014; Baez et al., 2022), much remains unknown about the regulation of respiratory enzymes of microorganisms from more extreme origins. For instance, the oxic layer of an exotic environment such as a hot spring is confined to the uppermost 1–2 cm (Revsbech and Ward, 1984; Jørgensen and Nelson, 1988) and can undergo diurnal variations (van der Meer et al., 2005). This environment selects microbes with a branched respiratory chain to adapt to fluctuating oxygen levels. In the case of microbes from hot springs, a compounding challenge is the combined effects of high temperature and extreme pH. The natural habitat of the obligate aerobic thermoalkaliphile *Caldalkalibacillus thermarum* TA2.A1 is an alkaline hot spring with a pH of 10 and 65°C, Mount Te Aroha, New Zealand (Peddie et al., 1999). To cope with the broad spectrum of oxygen concentrations it faces in its native environment, *C. thermarum* has a highly branched proton-mediated ETC (de Jong et al., 2020).

**Figure 1:**
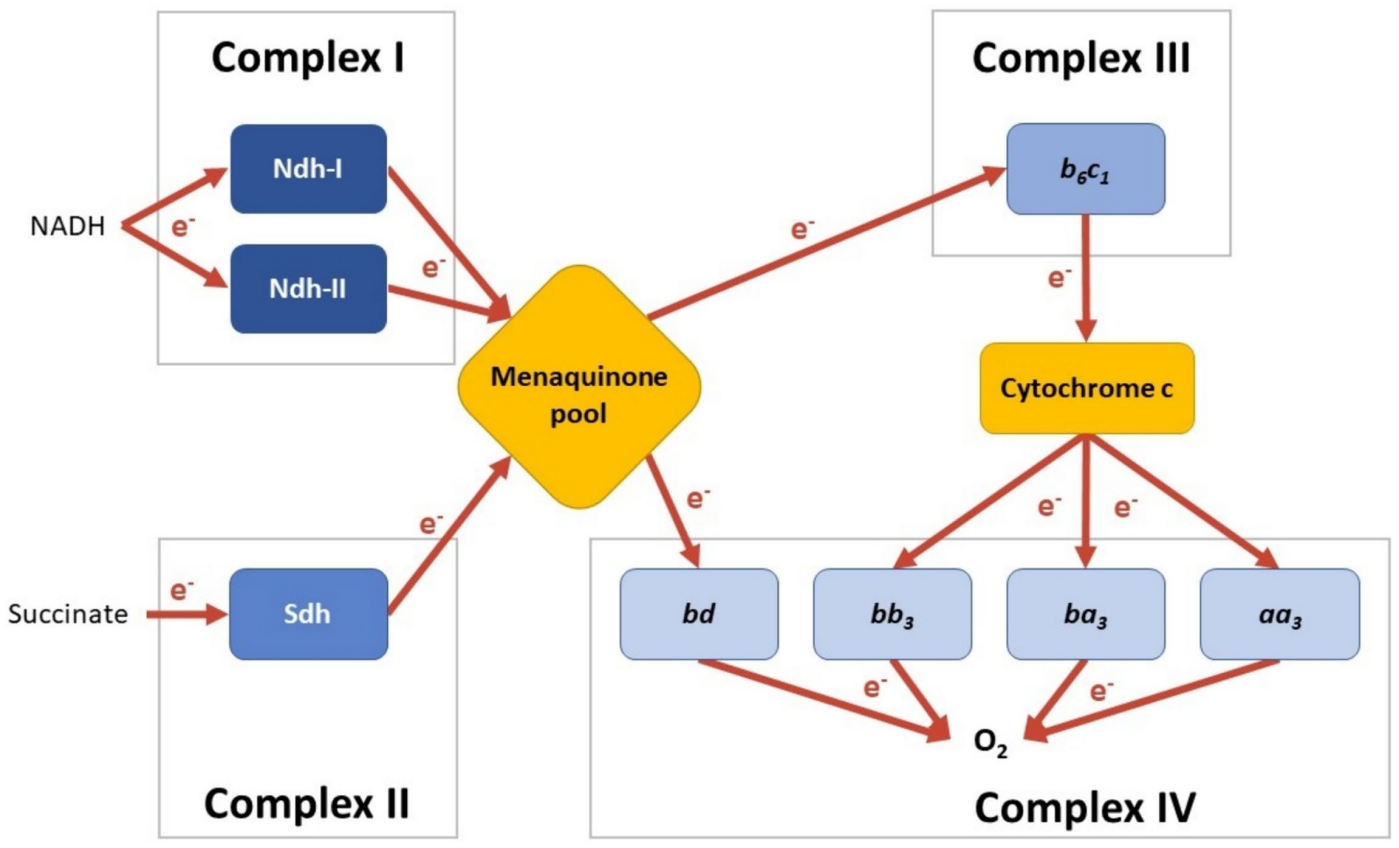
Schematic of the menaquinone-mediated *C. thermarum* TA2.A1 electron transport chain (ETC). C. Enzymes are colored per reaction type, and the electron flow is visualized with arrows. Note that currently, no condition is known under which all options are expressed simultaneously.

*C. thermarum* has a putative type I (Ndh-I) and a type II (Ndh-II) NADH dehydrogenase (de Jong et al., 2020). Ndh-I from aerobic bacteria are large proton-pumping multi-subunit integral membrane proteins that regenerate NAD^+^ from NADH (Figure 1; Brandt, 2006). In contrast, Ndh-II are small single-subunit peripheral membrane proteins with the same regeneration function but without pumping protons (Figure 1; Melo et al., 2004). The *C. thermarum* Ndh-II is biochemically well-described and is capable of rapid NADH turnover in a membrane environment (Godoy-Hernandez et al., 2019). *C. thermarum* also harbors a succinate dehydrogenase (Sdh) and a putative fumarate reductase (Figure 1; fumarate reductase not depicted; Hederstedt and Rutberg, 1981; Van Hellemond and Tielens, 1994). Little is known about either enzyme in *C. thermarum;* however, Sdh activity has been measured in purified *C. thermarum* membranes (McMillan et al., 2009).

There is a putative hybrid electron-pair splitting Complex III menaquinol:cytochrome *c* oxidoreductase *b*_6_*c*_1_ complex (Cyt. *b*_6_*c*_1_; de Jong et al., 2020). Cyt. *b*_6_*c*_1_ is a hybrid cytochrome with high sequence similarities between the cytochromes *bc*_1_ and *b*_6_*f*, both of which are integral membrane protein complexes and are found in mitochondrial and cyanobacterial electron transport chains, respectively. Cyt. *b*_6_*c*_1_ serves to transfer electrons from menaquinone to cytochrome *c* (Figure 1; Sone et al., 1996; Saribas et al., 1999). *C. thermarum* also boasts four putative terminal oxidases, all integral membrane proteins (de Jong et al., 2020). Cytochrome *aa*_3_ (Cyt. *aa*_3_), cytochrome *ba*_3_ (Cyt. *ba*_3_), and cytochrome *bb*_3_ (Cyt. *bb*_3_) rely on cytochrome *c* as an electron source (see Figure 1). Cyt. *aa*_3_ is known as an ‘aerobic oxidase’, typically being expressed under aerobic growth conditions (Von Ballmoos et al., 2012). A model Cyt. *aa*_3_ from *Rhodobacter sphaeroides* pumps protons at an efficiency of 0.7 H^+^/electron (Hosler et al., 1992). Cyt. *ba*_3_ and Cyt. *bb*_3_ are not as biochemically well-described as Cyt. *aa*_3._ The *Thermus thermophilus* Cyt. *ba*_3_ and *bb_3_* both pump protons, but with a lesser efficiency of 0.5 proton per electron transferred to the catalytic site (Pitcher and Watmough, 2004; Von Ballmoos et al., 2012). This leads to speculation that they may differ in oxygen affinity in *C. thermarum*. In support of this, Cyt. *bb*_3_ oxidase allows human pathogens to colonize anoxic tissues and agriculturally important microbes involved in nitrogen fixation (Pitcher and Watmough, 2004). An *Escherichia coli* homolog of the final terminal oxidase, Cytochrome *bd* (Cyt. *bd*), takes its electrons straight from the quinone pool rather than through cytochrome *c* (Figure 1; Asseri et al., 2021), so it is likely the same occurs in *C. thermarum*. In addition, in contrast to the other three oxidases, Cyt. *bd* does not pump any protons.

Finally, completing the ensemble is a proton-coupled F_1_F_o_-ATP synthase (Cook et al., 2003; McMillan et al., 2007). The *C. thermarum* enzyme is a large multi-subunit integral membrane protein complex that natively unidirectionally synthesizes ATP (McMillan et al., 2007, 2016). The enzyme is highly adapted to function in alkaline pH conditions to be able to recapture protons for ATP synthesis and to be irreversible (Keis et al., 2006; McMillan et al., 2007, 2016; Krah et al., 2023).

Previous investigations into the membrane proteome of *C. thermarum* in the stationary phase of batch cultivation found that Ndh-I, Ndh-II, Sdh, Cyt. *b*_6_*c*_1_, Cyt. *aa*_3_, Cyt. *ba*_3,_ and the ATP synthase were expressed (de Jong et al., 2023). However, the regulation of the *C. thermarum* TA2.A1 ETC as a response to differing oxygen availability has never been researched before. To study respiratory regulation as a function of oxygen availability in *C. thermarum* TA2.A1, cultivation in chemostat cultures is required. The use of chemostat bioreactor operation is preferred over other methods as it provides the most replicable results and because it allows precise dosing of oxygen. The term ‘aerobiosis’ was initially coined for *E. coli* to quantify the microaerobic range using acetate production as a benchmark (Alexeeva et al., 2002). Presumably, in *C. thermarum,* the Ndh-I, Sdh, Cyt. *b*_6_*c*_1_, and Cyt. *aa*_3_ respire the bulk of the electron potential under fully aerobic conditions as this is the most energy-efficient combination available. The primary aim of this study assesses whether that expectation is correct and to test the hypothesis that Ndh-II and the alternative terminal oxidases replace the most energy-efficient respiratory proteins under oxygen limitation. Whole-cell proteomics can then be used to pinpoint the enzymes within the ETC responsible for respiration at every level of aerobiosis.

Distinctive to alkaliphiles is their inverted proton gradient, whereby the internal pH is lower than that of the external environment. This makes proton homeostasis in alkaliphiles a subject that has been studied intensively before (Ito et al., 1997; Aono et al., 1999; Padan et al., 2005; Swartz et al., 2005; Slonczewski et al., 2009). As such, a secondary aim of this study is to determine whether the response to oxygen scarcity is comparable to model organisms. If our hypothesis that *C. thermarum* switches to its alternative ETC proteins proves correct, pH homeostasis must adapt as a result of that. Potential knock-on effects of decreased proton translocation on pH homeostasis in *C. thermarum* TA2.A1 might occur and are an additional area of interest. Finally, this study also aims to pinpoint overarching metabolic changes and benchmark them against model organisms. The multifaceted approach will result in a comprehensive insight into the response of *C. thermarum* to varying oxygen availabilities.

## Materials and methods

### Cultivation medium

*C. thermarum* TA2.A1 was cultivated in a medium adapted from an earlier study on the same organism (McMillan et al., 2009). The medium composition in this study was (in g L^−1^) as follows: tryptone peptone (Difco), 10.0; sucrose, 10; NaHCO_3_, 9.0; Na_2_SO_4_, 0.5; K_2_HPO_4_, 0.2; (NH_4_)_2_SO_4_, 0.1; MgSO_4_·7 H_2_O, 0.1; MnCl_2_·4 H_2_O, 5.0 × 10^−5^; ZnSO_4_, 1.4 × 10^−5^; Na_2_MoO_4_·2 H_2_O, 1.2 × 10^−5^. In the case of cultivation in a bioreactor, 0.25 mL L^−1^ of Antifoam C (Sigma-Aldrich, Missouri, United States) was added to the medium. Medium without sucrose was autoclaved at 121°C for 15 min. The pH of the medium was set to 9.5 before autoclaving. Sucrose was autoclaved separately at 110°C in a 50% (w/v) concentrate and added aseptically afterward; basal medium was concentrated during preparation accordingly.

### Bioreactor operation

A 3.0 L jacketed bioreactor (Applikon Biotechnology, Netherlands) was used with two Rushton impellers for stirring at 800 rpm, controlled by an ADI 1012 (Applikon Biotechnology, Netherlands). The working volume inside the reactor was kept at 1.0 L by continuous addition of medium and continuous removal of effluent. After an initial batch phase (see below), reactors in this study were operated in a chemostat, with a dilution rate of D = 0.1 h^−1^. The temperature was kept at 65°C by a thermostat, an Ecoline Staredition E 300 (Lauda, Germany). The reactor was sparged with an air-N_2_ mix; to reach the desired aerobiosis level, this mix was altered each time (Table 1). Off-gas was cooled using an RM6S Refrigerated Circulating Bath (Lauda, Germany) as cryostat, and the composition thereof was subsequently measured using an NGA 2000 off-gas multiplexer (Rosemount Inc., Minnesota, USA). The pH was kept stable at 9.5 by the automatic addition of either 2 M H_2_SO_4_ or 2 M NaOH, controlled by an ADI 1030 Bio Controller (Applikon Biotechnology, Netherlands). Dissolved oxygen was measured with an AppliSens DO probe (Applikon Biotechnology, Netherlands), and the pH was measured using an AppliSens pH+ probe (Applikon Biotechnology, Netherlands), both with a length of 235 mm.

**Table 1:** Required mix of compressed air and nitrogen gas to sparge with desired O_2_ concentrations in the bioreactor.

Oxygen level in gas inlet (%)	Air (L_n_ min^−1^)	Nitrogen gas (L_n_ min^−1^)
4.2	0.200	0.800
2.05	0.100	0.900
1.05	0.050	0.950
0.42	0.020	0.980
0.25	0.012	0.988

Five conditions were used and can be identified by their final O_2_ concentration in the gas inlet. Note these are only the O_2_ concentrations in the gas inlet. Regardless of the O_2_ concentrations in the inlet gas, the dissolved oxygen concentration was always 0%.

The inoculum for each bioreactor replicate was started by thawing a fresh ±1.6 mL glycerol stock of *C. thermarum* TA2.A1 (frozen at −80°C). All stocks used in this research line originate from the same batch. Thawed cells were reconstituted in a 500 mL round bottom shake flask, with 100 mL working volume, at 65°C and 140 rpm for 18 h. Then, 2% of this culture was transferred to a fresh shake flask containing 100 mL medium and again cultivated for 18 h. The resulting culture was used entirely as reactor inoculum. In the reactor, a batch phase of roughly 8 h followed inoculation, after which the reactor was set to chemostat. Samples were taken periodically for optical density and dry weight measurements; the supernatant was kept at −20°C for acetate and sucrose concentration determination at a later stage. Steady state was assumed when off-gas profiles, OD_600_, and dry weight measurements were stable for three consecutive retention times. Aseptic conditions were maintained throughout the cultivation procedure. All aeration levels tested were biologically duplicated.

### Analytical methods

Optical density measurements were performed at a wavelength of 600 nm (OD_600_) with a 1 cm light path length in a Biochrom Ultrospec 2,100 Pro UV Vis Spectrometer (Amersham, United Kingdom). For dry weights, a 10 mL sample was filtered over a pre-dried 0.20 μm Supor® PES Membrane Disc Filter (Pall, New York, United States) and dried at 105°C; the filter was weighed before sample addition and after drying for >1 h. Acetate was measured using high-performance liquid chromatography (HPLC). HPLC analysis was performed with 1.5 mmol L^−1^ H_3_PO_4_ as eluent at a flow of 0.75 mL min^−1^. The compounds were separated over an Aminex HPX-87H column (BioRad, California, United States) at 59°C and thereafter detected by a refractive ERC RefractoMax 520 (ERC Inc., Japan). Sucrose was measured using the GOPOD d-Glucose Assay Kit (Megazyme, Ireland), with the addition of 160 mg L^−1^ Grade VII Invertase (300 U mg-1 from *Saccharomyces cerevisiae*; Sigma-Aldrich, Missouri, United States) to the reaction mix. Except for the addition of the invertase enzyme to convert sucrose into glucose, the manufacturer’s specifications were followed; the same UV Vis detector was used as for optical density measurements. Analytical measurements were conducted in duplicate.

### Protein extraction and proteomics

For proteomics, at the end of each condition, a 100 mg sample (wet weight) was taken and washed in ice-cold phosphate-buffered saline. This sample was flash-frozen in liquid nitrogen and stored thereafter at −80°C until analysis. For analysis, the protocol of which was based on extensive research (den Ridder et al., 2022, 2023), the samples were thawed. Then, 22.2 ± 0.5 mg sample was dissolved in 0.175 mL 1 M triethylammonium bicarbonate buffer in a LoBind tube (Eppendorf, Germany) and thereafter treated exactly according to the ‘whole-cell’ procedure in our previous study (de Jong et al., 2023). This is also true for shotgun proteomic analysis up to the point where the results were exported as ‘proteins.csv’ files (Kleikamp et al., 2021; Pabst et al., 2021).

### Data processing and visualization techniques

The proteomic data in this study were categorized in two different ways: by Kyoto Encyclopedia of Genes and Genomes (KEGG) modules and the membrane found in our previous study (de Jong et al., 2023), with some proteins falling in both categories. All analyses were performed in Python. Modules were retrieved from the KEGG database using the ‘*requests*’ library; the identifier for *C. thermarum* TA2.A1 genome is ‘*cthu*’. From the absolute abundances in ‘proteins.csv’, log2 ratios were calculated to equally assess up- and downregulation under the various conditions; 4.2% O_2_ was used as a reference. The resulting data were collected in a single Excel file. This Excel file, containing the proteomics data of each condition and replicate, was subsequently loaded in a *pandas* dataframe and the script was automatically checked which proteins of a certain module were discovered in the shotgun proteomics analysis. In the case of this study, if six or more proteins were found for a specific module in each condition, data were stored in a separate Excel file to make a boxplot. The boxplot was made using the ‘*matplotlib.pyplot*’ library, and settings were adjusted to show the average, whiskers, and outliers. Module names were added manually. For the heat maps of the short-chain fatty acid module (Supplementary Figure S1) or of membrane proteins of interest, the ‘*visuz*’ package of the ‘*bioinfokit*’ library was used. Fake data were added to ensure a log_2_ ratio of zero was always the center.

## Results and discussion

### An overview of the adaptation to oxygen limitation by *C. thermarum* TA2.A1

The goal of this study was to assess the regulation of the *C. thermarum* ETC, uncover potentially alkaliphile-specific adaptation, and assess overarching metabolic changes under varying oxygen availabilities. The first objective was to find the required oxygen level in the gas inlet at which oxygen would become limiting (i.e., dissolved oxygen = 0 at steady state). Preparatory chemostat cultivations were performed at a broader range than reported here; the highest aeration level at which oxygen limitation was detected was 4.2% O_2_ in the gas inlet; the lowest oxygen level at which growth was detected was 0.25% O_2_ (Supplementary Figures S2, S3). In all of these conditions, the dissolved oxygen concentration was zero, meaning consumed oxygen was limited by the oxygen transfer capacity. For the purpose of this report, *C. thermarum* was grown with five different oxygen levels in the gas mix: 4.2, 2.05, 1.05, 0.42, and 0.25%. As described above, N_2_ gas was added to keep gas flow at 1.0 L_n_ min^−1^. The biomass-specific substrate consumption rate q_s_ barely varied at 0.42% O_2_ and above: 29.3 mmol_s_ Cmol_x_^−1^ h^−1^ ≤ q_s_ ≤ 38.5 mmol_s_ Cmol_x_^−1^ h^−1^. At the 0.25% O_2_, an increase was measured: q_s_ = 51.0 mmol_s_ Cmol_x_^−1^ h^−1^. While all bioreactors operated in oxygen-limited conditions, acetate production only occurred at 2.05% O_2_ and lower mole fractions. The biomass-specific acetate production rate q_ac_ varied between 44.2 mmol_ac_ Cmol_x_^−1^ h^−1^ and 123.6 mmol_ac_ Cmol_x_^−1^ h^−1^. Although acetate production generally increased with decreasing oxygen, no linear trend was observed, unlike what is described for *E. coli* (Alexeeva et al., 2002). From a basic physiological perspective, *C. thermarum* follows the same strategy to combat oxygen limitation, which is to supplement its failing aerobic respiration with increasing amounts of substrate-level phosphorylation provided by partial acetate fermentation.

**Figure 2:**
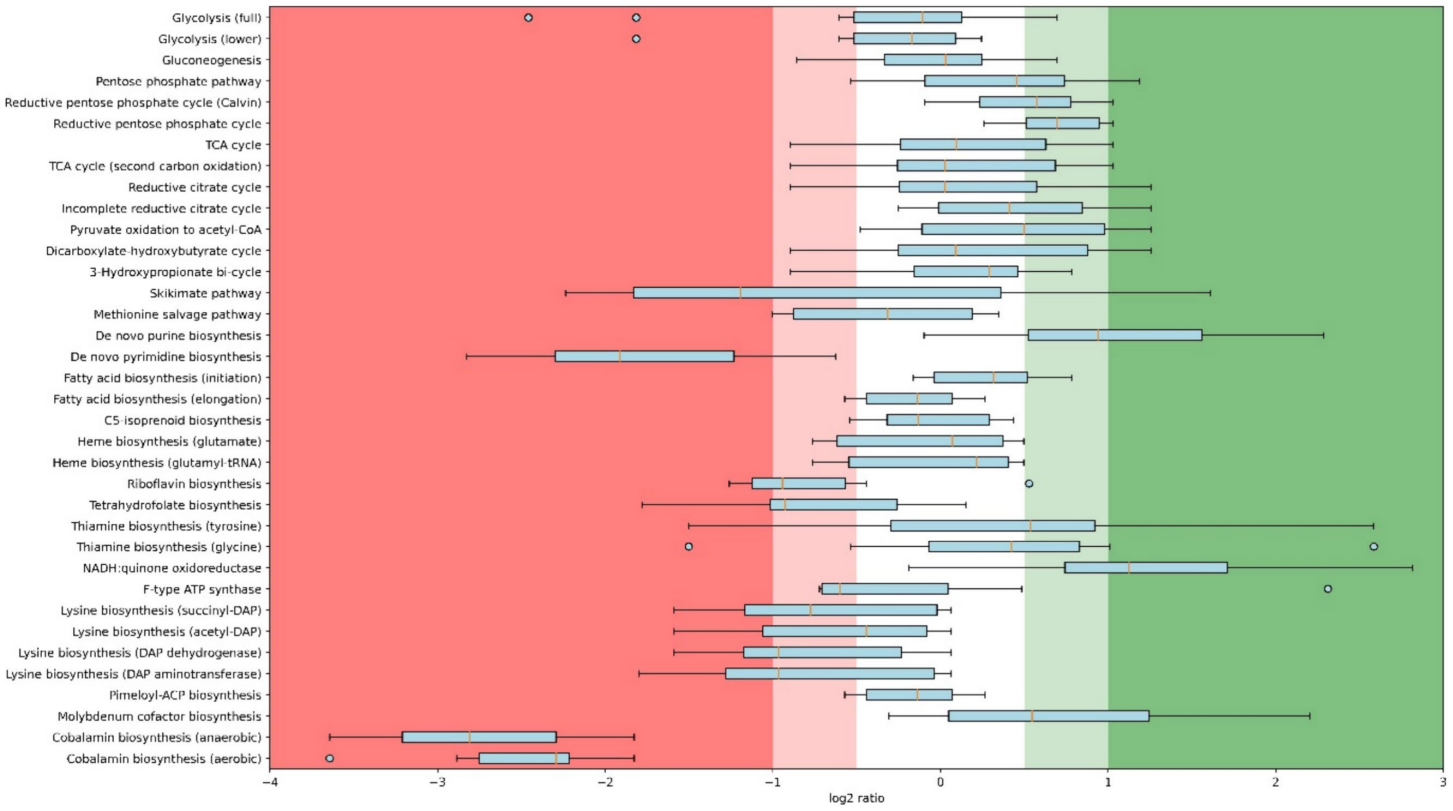
Box plot with whisker and outliers comparing proteomics results of 4.2% O_2_ (highest level) and 0.25% O_2_ (lowest level) chemostats per pathway. Regulation is calculated as log_2_ ratios; 0.25% O_2_ relative to 4.2% O_2_. Displayed pathways are KEGG modules of which at least 2 unique peptides of 6 or more proteins are detected in both conditions. Module names are as descriptive as possible, underlying KEGG identifiers can be found in Supplementary Table S1, and the underlying proteomics data is publicly available (see below).

Whole-cell proteomics was performed for both replicates of each condition. To determine on a more detailed level whether the regulation under oxygen-limited conditions is comparable to model organisms, proteomics of the highest (4.2% O_2_) and lowest (0.25% O_2_) levels were compared. Figure 2 shows this regulation per pathway, calculated as log_2_ ratios. As stated above, *C. thermarum* uses a different strategy compared to model organisms such as *E. coli* and *S. cerevisiae* to move from respiration to fermentation. The discrepancy between this study and the model organism study stems from the aforementioned stable *q_s_* in this study and the fact that *C. thermarum* cannot grow under absolute anaerobic conditions. Together with the *q_s_*, glycolysis remains stable in *C. thermarum*, while upregulation of glycolysis is generally observed in *E. coli* and *S. cerevisiae* (de Groot et al., 2007; Steinsiek et al., 2011; Ederer et al., 2014) to ensure enough carbon is available to ferment. The partial fermentation of *C. thermarum* to acetate likely stems from the carbon flux being diverted from the TCA cycle to acetate production, without the requirement of significant proteomics reallocations over a whole pathway. Proteomics data show an increase in enzymes responsible for acetate production, Pta and Ack (Supplementary Figure S1), to facilitate the increased carbon flux through that route. Few previous studies tried to also assess anabolic proteomics changes, yet some interesting observations deserve discussion. A few pathways are entirely up- or downregulated more than 2-fold under 0.25% O_2_ relative to 4.2% O_2_ (i.e., having a log_2_ ratio greater than −1 or + 1). Three pathways stand out as being multifold downregulated as a whole: *de novo* pyrimidine biosynthesis and both cobalamin biosynthesis pathways. Enzymes of pyrimidine biosynthesis are known to be 6–20 times more active under anaerobic conditions, as was first reported in *Staphylococcus aureus* (McIllmurray and Lascelles, 1970). A similar increase in enzymatic activity in *C. thermarum* under diminished oxygen conditions should decrease the production requirement of pyrimidine synthesis proteins, causing the observed downregulation. Cobalamin can be produced both aerobically and anaerobically, and *C. thermarum* contains both pathways. Both of these pathways are downregulated more than 4-fold. Decreased cobalamin production is because cobalamin was involved in protection against reactive oxygen species (ROS) as has been demonstrated in the acidophile *Leptospirillum* Group II *CF*-I (Ferrer et al., 2016). Having this function in *C. thermarum* next to its various regular functions would explain the decreased requirement when oxygen becomes limiting (Neil and Marsh, 1999; Raux et al., 2000). Two modules are completely upregulated more than 2-fold: NADH:menaquinone oxidoreductases, which will be discussed later, and *de novo* purine biosynthesis.

The upregulation of purine biosynthesis in anaerobiosis has been previously shown in *S. cerevisiae* (de Groot et al., 2007). A plausible cause for the upregulation of purine biosynthesis proteins is that its regulatory enzyme, a glutamine 5-phoshophoribosyl-1-pyrophosphate amidotransferase, is oxygen sensitive (Smith et al., 1994). Whether any post-translational modifications exist to prevent intracellular buildup of purines is unknown; however, it is highly likely.

### Unraveling the role of the many respiratory enzymes

**Figure 3:**
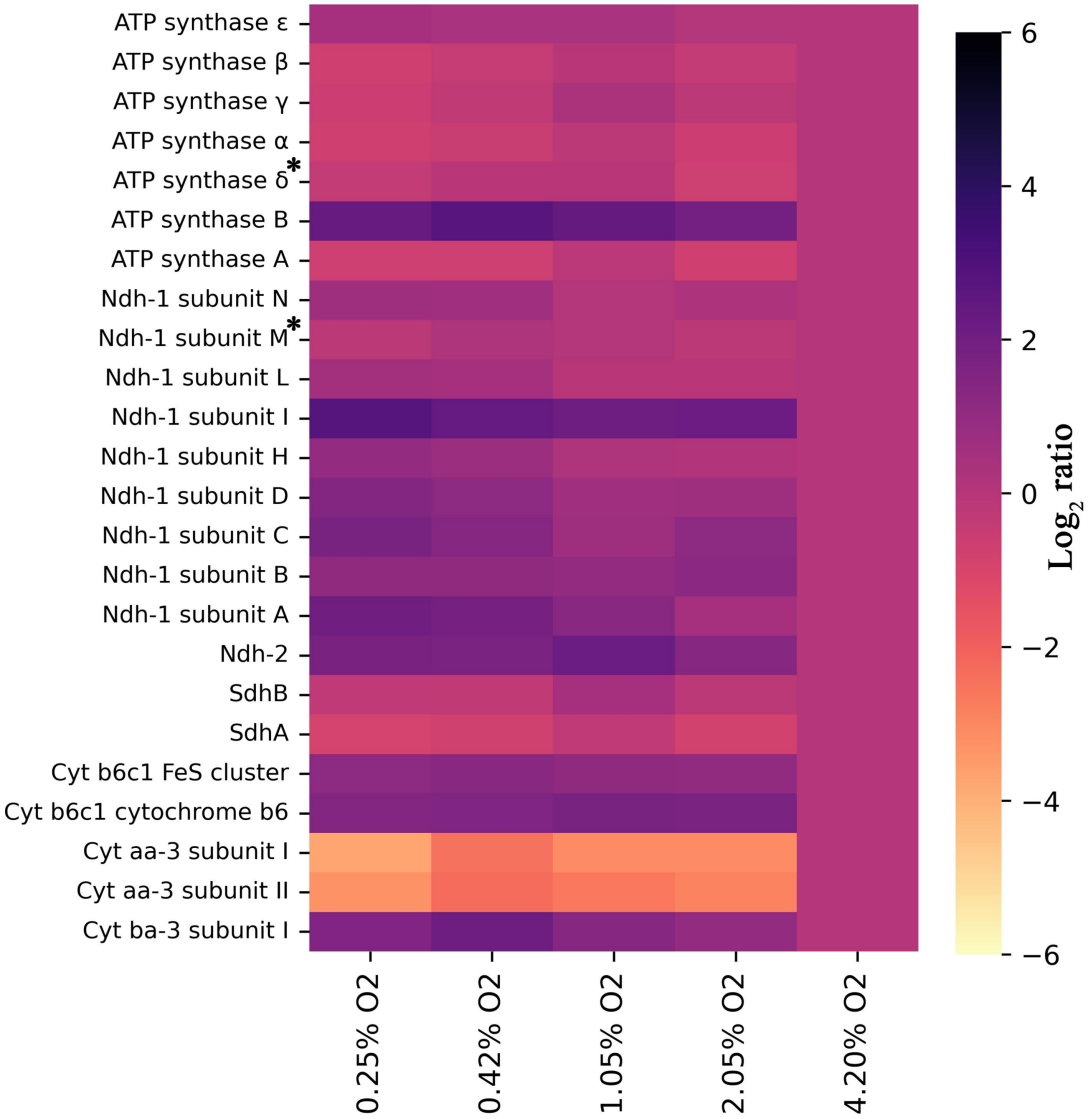
Heat map showing log_2_ ratios of all detected subunits of the ETC for each condition, provided two or more unique peptides were detected, relative to 4.2% O_2_. All trends have a significance of *p* < 0.05, except those denoted with an asterisk. For ATP synthase subunit *δ*, the *p* = 0.058, and for Ndh-1 subunit M, the *p* = 0.150. For all others, the exact significances and the corresponding protein number can be found in Supplementary material S1 and the locus tags in Supplementary Table S1.

As mentioned in the previous section, and contrary to expectations, a higher abundance of NADH:menaquinone oxidoreductases was detected at 0.25% O_2_ compared to 4.2% O_2_. If assessing per subunit though, as detailed in Figure 3, the upregulation is not as stark for Ndh-I. Rather, a few subunits, namely, subunits A, C, and I, have log_2_ ratios exceeding 1.5, while the other are closer to 0.5–1.0. It is curious that the subunits are differentially regulated to an extent. Conclusions are somewhat more definitive with Ndh-II, which has a log_2_ ratio of 1.8 at 1.05% O_2_ (Figure 3). When compared to *E. coli*, in which solely Ndh-II has been shown to be *upregulated* and Ndh-I *downregulated* when oxygen becomes limiting, this result is surprising (Steinsiek et al., 2011). This result puzzles even more considering the type II NADH dehydrogenase of *C. thermarum* has a higher maximum specific activity than its homolog in *E. coli* (Björklöf et al., 2000; Heikal et al., 2014; Blaza et al., 2017; Godoy-Hernandez et al., 2019). Normally, in *E. coli*, the switch to acetate during decreased aerobiosis is coupled to concomitant ethanol fermentation (Alexeeva et al., 2002). This is because while acetate production provides ATP, ethanol is required to regenerate NAD^+^. *C. thermarum* is unable to make ethanol. The inability of *C. thermarum* to produce ethanol could explain the higher Ndh-I abundance at lower oxygen levels, though the authors would reason completely switching to Ndh-II would be more efficient. More research is required to fully explain this counterintuitive behavior.

The succinate dehydrogenase (Sdh) and the F_1_F_o_-ATP synthase were downregulated, following the same protein presence patterns detailed for *E. coli* (Figure 3; Ederer et al., 2014). Downregulation of Sdh is another contributing factor to the observed acetate production. The inability to fully utilize the TCA cycle dictates that pyruvate should be consumed by another pathway. *E. coli* does not have any genes encoding for a Complex III, but *S. cerevisiae* does. In a previous yeast study (de Groot et al., 2007), its cytochrome *bc*_1_ complex is reported to be downregulated but sadly not to what extent. In this study, *C. thermarum*’s Complex III, the cytochrome *b*_6_*c*_1_ complex, is slightly upregulated at lower O_2_ levels. This observation could be related to the aforementioned upregulation of Ndh-I. Another potential explanation for Ndh-I upregulation and F_1_F_o_-ATP synthase repression at low O_2_ is that the intracellular pH may be increased to keep the redox homeostasis. Babauta et al. (2012) suggested intracellular pH could modify redox potential by virtue of low oxygen concentration decreasing intracellular ROS concentration (Baez and Shiloach, 2013) and increasing NADPH content (Baez and Shiloach, 2017) and, in this theory, would be very subtle, because pH in itself is also a strong regulatory mechanism in the context of alkaliphile. In this context, it is unsurprising that the epsilon subunit of the *C. thermarum* F_1_F_o_-ATP synthase, an enzyme with the capability of proton pumping, is heavily regulated by pH (Krah et al., 2023).

Two types of terminal oxidases were detected in this experiment, in fact in all conditions: Cyt. *aa*_3_ and Cyt. *ba*_3_. As expected, Cyt. *aa*_3_ is downregulated when oxygen becomes limiting, while Cyt. *ba*_3_ is upregulated (Figure 3). The main difference between these two enzymes is that Cyt. *aa*_3_ allows for the translocation of 2 H^+^ per 2 e^−^, while Cyt. *ba*_3_ only translocates 1 H^+^ per 2 e^−^. As described in the introduction, a similar pattern is observed in *E. coli*. This organism switches from its cytochrome *bo*_3_ (pumping 1H^+^/electron), through cytochrome *bd* (pumping 0H^+^/electron) and finally to cytochrome *bd*-II (pumping 0H^+^/electron) under increasing anaerobiosis (Steinsiek et al., 2011, 2014). Although the abundance of Cyt. *ba*_3_ in this study starts to tail of below 0.42% O_2_, no other terminal oxidase was detected. This is likely due to the hydrophobicity of both proteins and accessibility to proteolytic digestion, a major challenge in membrane proteomics. Regardless, based on our findings of *aa*_3_ and *ba*_3_, it seems most likely that Cyt. *bb*_3_ or Cyt. *bd* takes over responsibility for oxygen electron transfer at oxygen concentrations less than 0.25%. Cyt. *bb*_3_, as described in the introduction, allows human pathogens to colonize anoxic tissues and translocates 1 H^+^ per electron pair, but possibly with a higher affinity since it is expressed at lower O_2_ concentrations. Cyt. *bd* is also known to be used at low oxygen conditions in *E. coli* (Steinsiek et al., 2011; Ederer et al., 2014).

### Is a low oxygen adaptation specific for alkaliphiles?

**Figure 4:**
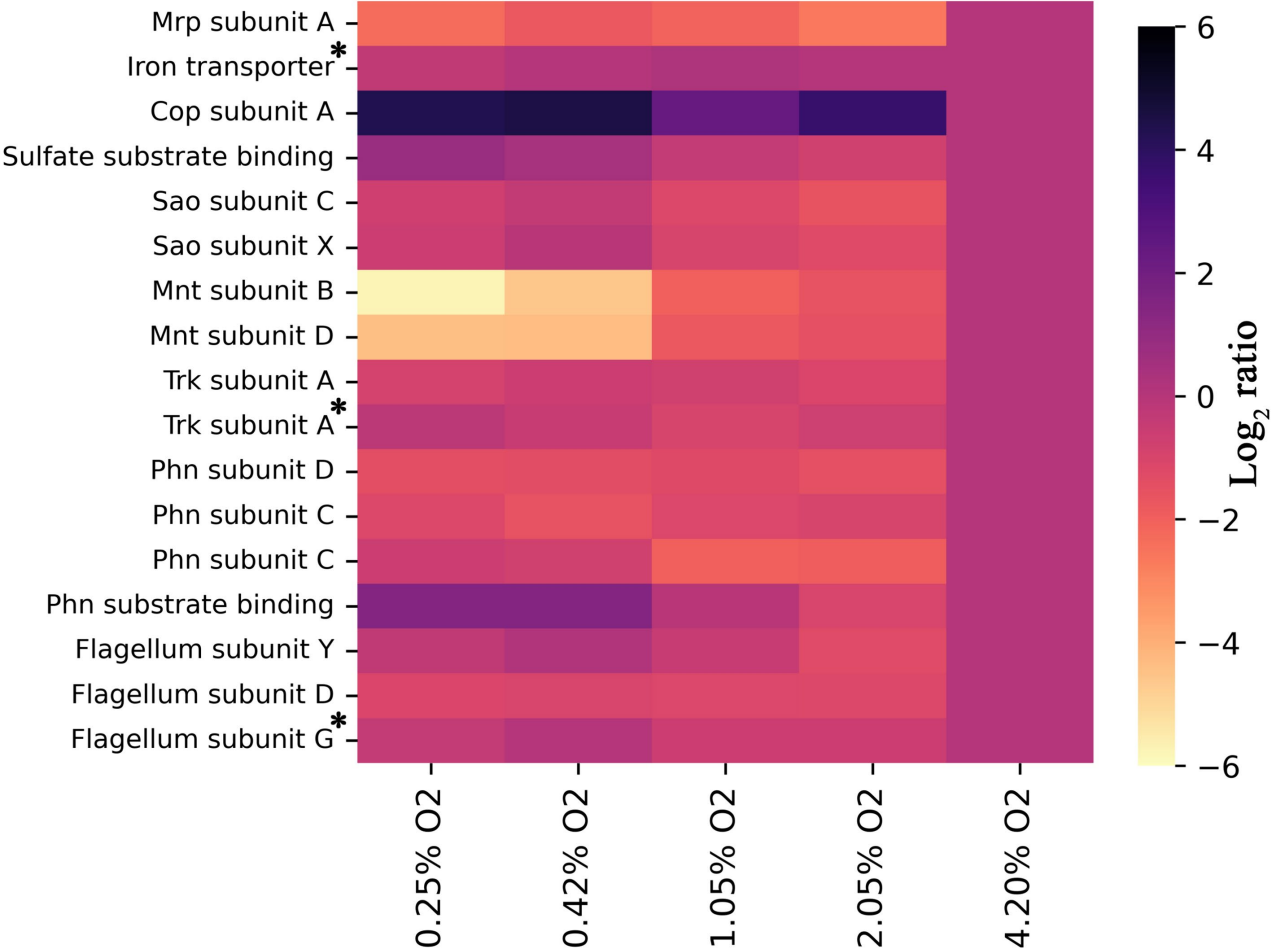
Heat map showing log_2_ ratios of all detected subunits of known transporters of inorganic compounds for each condition, provided two or more unique peptides were detected, relative to 4.2% O_2_. All trends have a significance of p < 0.05, except those denoted with an asterisk. For the iron transporter *p* = 0.255, for the Trk subunit A *p* = 0.053, and for the Flagellum subunit G *p* = 0.101. For all others, the exact significances and the corresponding protein number can be found in Supplementary material S1 and the locus tags in Supplementary Table S1.

In addition to the respiratory chain which is obligately coupled to oxygen, other membrane processes also adapt at different oxygen availabilities (Figure 4). Figure 4 shows the log_2_ ratios for transporters detected in this study and our previous study on unraveling the membrane proteome of this organism (de Jong et al., 2023). There is a striking 6-fold upregulation of CopA, the ATP-coupled copper export mechanism linearly coupled to oxygen limitation (Figure 4). Copper can be toxic to cells, and in *Rubrivivax gelatinosus*, it has been demonstrated that CopA mutants have decreased cytochrome *c* oxidase activity (Azzouzi et al., 2013). The solubility of copper is lower at high pH than under the conditions used in *R. gelatinosus* (Cuppett et al., 2006), perhaps further exacerbating this issue. In addition to this, copper efflux might be induced in *C. thermarum* to protect iron–sulfur cluster enzymes from copper mis-metallation (Fung et al., 2013; Imlay, 2014). This is because the intracellular labile Fe^2+^ pool is higher under oxygen limitation, which leads to a net increase of Fe^2+^-Fur activity with concomitant iron-protein expression (Beauchene et al., 2017). This gains feasibility, especially considering the ionic radii of Fe^2+^ is 0.076 nm vs. Cu^2+^ 0.073 nm and Cu^1+^ at 0.077 nm (Cotton et al., 1999). Finally, another possible scenario is that the copper requirement of the cell decreases at lower oxygen levels, due to the decreased Cyt *aa*_3_ production, for which copper is required. While Cyt. *ba*_3_, also requiring copper, is produced as an alternative, the absolute amount of each individual enzyme can differ quite starkly; data in this study only show the regulation of proteins relative to themselves. A decreased content of heme-copper oxidases could increase the need for copper export and thus explain the observed pattern. In contrast, the manganese transport complex Mnt is downregulated 6-fold. Generally, manganese is required for living with oxygen (Horsburgh et al., 2002), explaining the lower requirement at low O_2_.

Aside from Mnt, the transporter Mrp is also significantly downregulated at lower O_2_ concentrations. The Mrp antiporter facilitates the export of Na^+^ coupled with the import of H^+^. This antiporter is considered a vital protein for alkaliphiles as it is one of the primary methods to regulate internal sodium *and* proton levels (Padan et al., 2001, 2005; Swartz et al., 2005). Data on the ETC show that proton export does not decrease to the extent that Mrp becomes useless. Another reason must drive the decreased need for sodium:proton antiport through Mrp. Since all other medium components remained the same, we especially expect sodium stress to remain as compelling for an alkaliphile such as *C. thermarum* TA2.A1 under low O_2_. Interestingly enough, the very first studies into *C. thermarum* TA2.A1 centered on the fact that substrate import (glutamate and sucrose) is mediated using sodium ions, not protons (Peddie et al., 1999, 2000). Though no *in vivo* nor *in vitro* data are reported regarding acetate export, the expectation is that this is also mediated by sodium ions (de Jong et al., 2020). Considering the high level of acetate production at low O_2_ levels, we hypothesize that the function of Mrp is—at least partly—replaced by the acetate exporter under these conditions. This would constitute a valuable addition to the field of alkaliphiles as no such regulation of Mrp, to the best of our knowledge, has been found previously.

## Conclusion

*C. thermarum* TA2.A1 was successfully cultivated within a microaerobic range in chemostats, demonstrating its low oxygen requirement. Oxygen limitation became evident near 4.2% O_2_ in the gas inlet, while the microorganism grew even at 0.25% O_2_ in the gas inlet. Acetate is produced concurrently with respiration to complement energy generation. Surprisingly, proteomics data showed that both NADH dehydrogenases are constitutively expressed under the conditions tested. It makes little sense that an Ndh-I is upregulated at low O_2_, pumping out protons, when the F_1_F_o_-ATP synthase that takes the protons back into the cell is downregulated under the same conditions. One potential explanation is that the intracellular pH should be increased at low O_2_ to keep the redox homeostasis functional.

Cyt. *aa*_3_ is the predominant contributor to the terminal oxidase pool at the highest oxygen level, while Cyt. *ba*_3_ takes over below 4.2% O_2_. Detection of either Cyt. *bb*_3_ or Cyt. *bd* was expected at 0.25% O_2_, given that the abundance of Cyt. *ba*_3_ started to decline. Neither Cyt. *bb*_3_ nor Cyt. *bd* was detected, but there is a probability that either or both are present at low oxygen levels. Proteomics analysis of membrane proteins needs to be further developed to fully use this methodology for the study of respiratory systems. Finally, a notable finding was a decreased abundance of the Mrp complex, considered a crucial cog in alkaliphilic sodium homeostasis. We hypothesized that this could be due to the presence of an acetate:sodium symporter, responsible for facilitating acetate export and thereby decreasing sodium stress.

## Data Availability

The datasets presented in this study can be found in online repositories. The names of the repository/repositories and accession number(s) can be found in the article/Supplementary material.
