# Supplementary material for: Quantitative proteomics reveals oxygen-induced adaptations in Caldalkalibacillus thermarum TA2.A1 microaerobic chemostat cultures
Source: Front Microbiol. 2024 Oct 28;15:1468929. doi: 10.3389/fmicb.2024.1468929 (PMC11551716; doi:10.3389/fmicb.2024.1468929)
Supplement: Supplementary file 1 [file Data_Sheet_1.docx]

# Supplementary Information

**
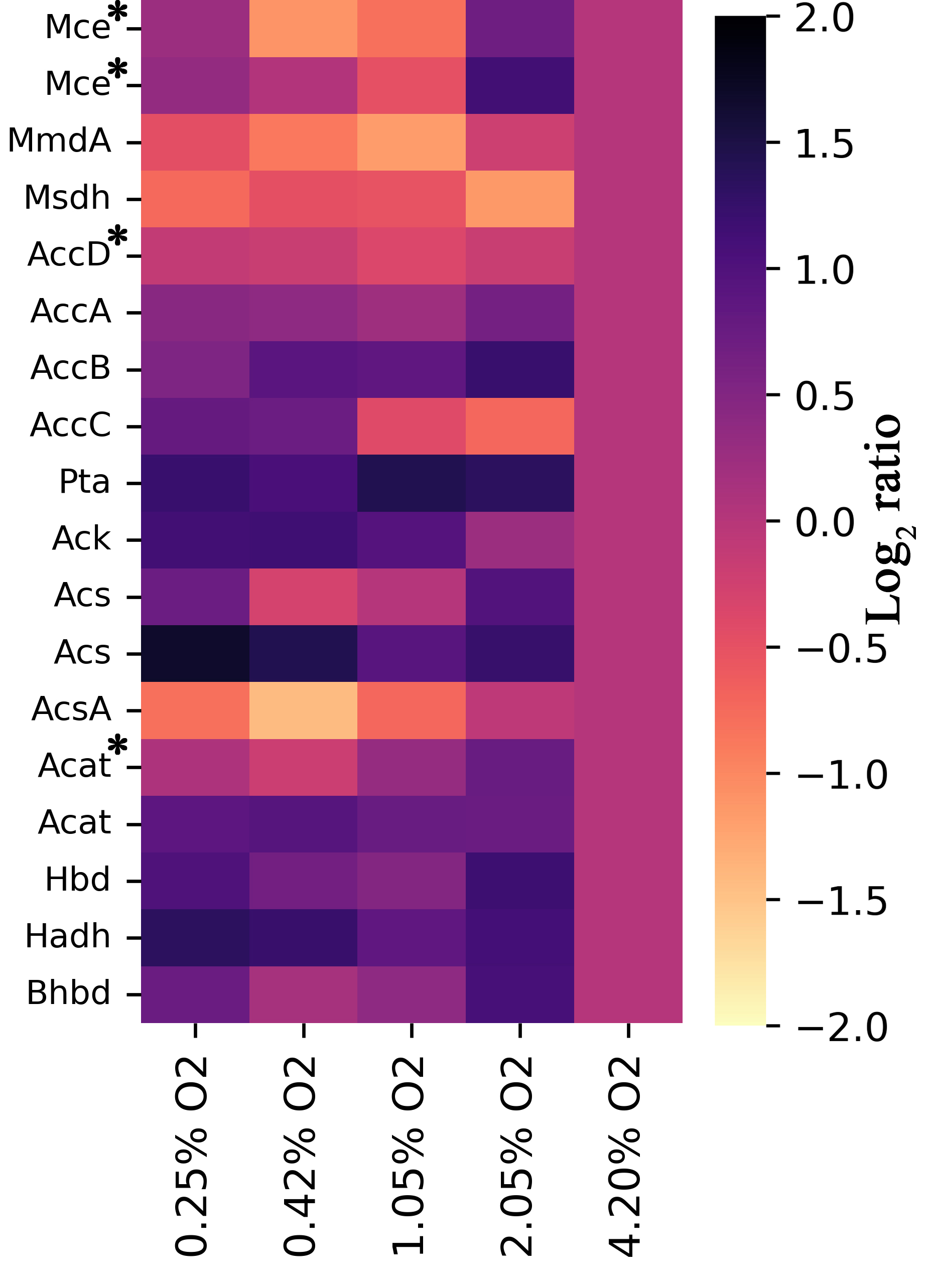
**

**Supplementary Figure I.** Heatmap depicting regulation of detected enzymes involved in the synthesis of short chain fatty acids. Of particular interest in this figure is the regulation of Pta and Ack, as is mentioned in the body of the text. All trends have a significance of p < 0.05, except those denoted with an asterisk. For the Mce genes, p = 0.061 and 0.140 respectively, for AccD p = 0.107 and for Acat p = 0.148. For all others, the exact significances and the corresponding protein number can be found in Supplementary File I, and the locus tags in Supplementary Table I.


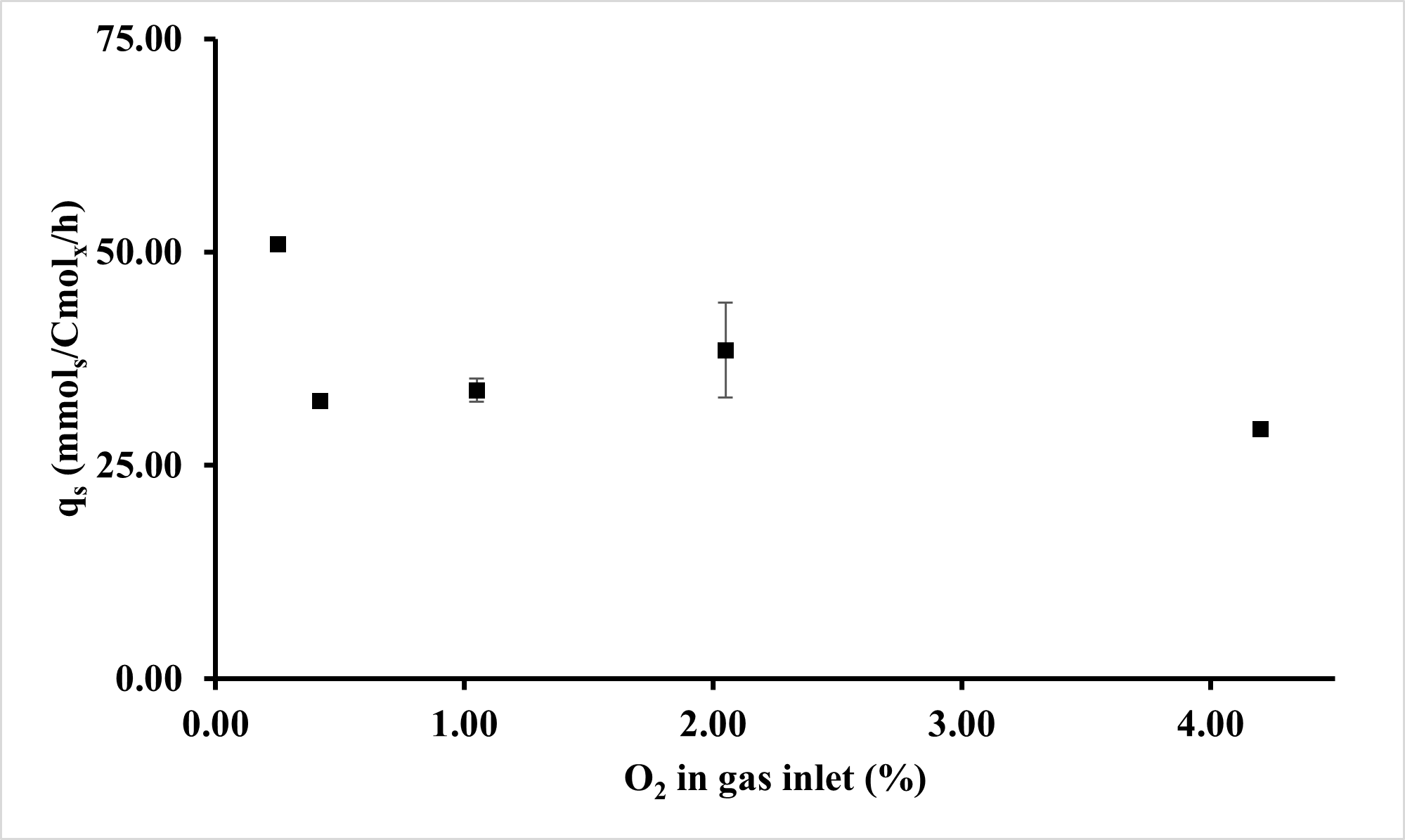


**Supplementary Figure II.** Biomass specific surose uptake rate (q_s_) data of *C. thermarum* chemostats. Raw data to obtain these values can be found in **Supplementary Table I**. All bioreactors were operated in biological duplicates and underlying analysis methods were replicated technically in addition. Error bars shown are the standard deviations over the entire sample set. The q_s_ was calculated with formula’s below.

$$q_{s}=\frac{R_{s}}{V_{L}\cdot c_{x}}$$

$$R_{s}=F_{out}\cdot c_{s,out}-F_{in}\cdot c_{s,in}$$

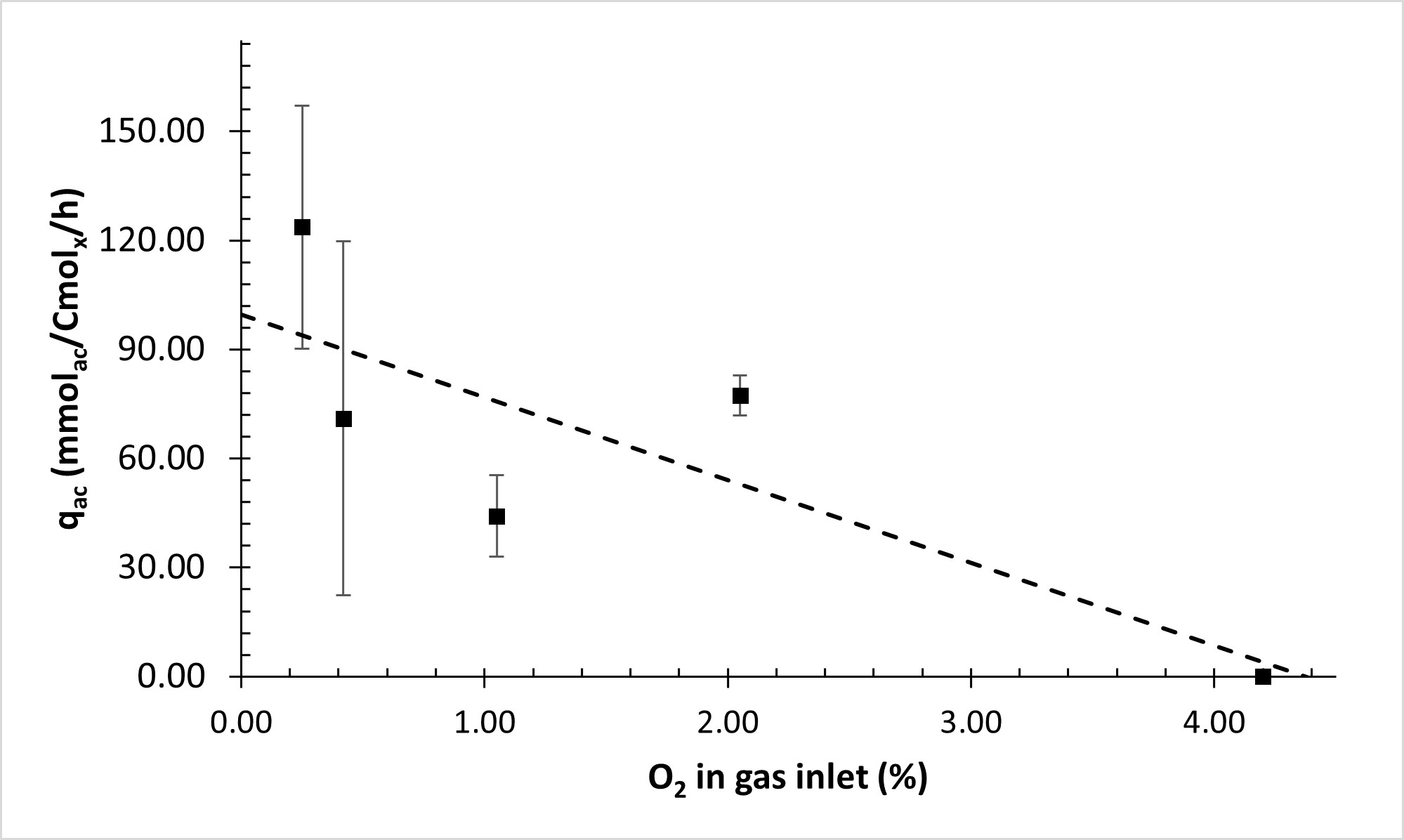


**Supplementary Figure III.** Biomass specific acetate production rate (q_ac_) data of *C. thermarum* chemostats. Raw data to obtain these values can be found in **Supplementary Table I**. All bioreactors were operated in biological duplicates and underlying analysis methods were duplicated technically in addition. Error bars shown are the standard deviations over the entire sample set. The q_ac_ was calculated with formula’s below.

$$q_{ac}=\frac{R_{ac}}{V_{L}\cdot c_{x}}$$

$$R_{s}=F_{out}\cdot c_{ac,out}-F_{in}\cdot c_{ac,in}$$

**Supplementary Table I.** Raw concentration values used to calculate the biomass specific rates of **Supplementary Figure II** and **Supplementary Figure III**.

| O_2_ in gas inlet (%) | c_x_ (g L^-1^) | c_s_ (mM) | c_ac_ (mM) |
| --- | --- | --- | --- |
| 4.2 | **0.725 ± 0.009** | **14.250 ± 6.219** | **0.000 ± 0.000** |
| 2.1 | **0.611 ± 0.066** | **19.552 ± 0.353** | **19.571 ± 0.730** |
| 1.05 | **0.332 ± 0.070** | **24.558 ± 0.799** | **6.452 ± 2.833** |
| 0.42 | **0.254 ± 0.019** | **17.510 ± 8.512** | **7.899 ± 5.714** |
| 0.25 | **0.190 ± 0.069** | **17.313 ± 6.399** | **8.843 ± 0.901** |

**Supplementary Table II.** KEGG Module names corresponding to names used in to **Figure 2**.

| **Pathway Identifier** | **KEGG Module** |
| --- | --- |
| Glycolysis (full) | M00001 |
| Glycolysis (lower) | M00002 |
| Gluconeogenesis | M00003 |
| Pentose phosphate pathway | M00004 |
| Reductive pentose phosphate cycle (Calvin) | M00165 |
| Reductive pentose phosphate cycle | M00167 |
| TCA cycle | M00009 |
| TCA cycle (second carbon oxidation) | M00011 |
| Reductive citrate cycle | M00173 |
| Incomplete reductive citrate cycle | M00620 |
| Pyruvate oxidation to acetyl-CoA | M00307 |
| Dicarboxylate-hydroxybutyrate cycle | M00374 |
| 3-Hydroxypropionate bi-cycle | M00376 |
| Skikimate pathway | M00022 |
| Methionine salvage pathway | M00034 |
| De novo purine biosynthesis | M00048 |
| De novo pyrimidine biosynthesis | M00051 |
| Fatty acid biosynthesis (initiation) | M00082 |
| Fatty acid biosynthesis (elongation) | M00083 |
| C5-isoprenoid biosynthesis | M00096 |
| Heme biosynthesis (glutamate) | M00121 |
| Heme biosynthesis (glutamyl-tRNA) | M00926 |
| Riboflavin biosynthesis | M00125 |
| Tetrahydrofolate biosynthesis | M00126 |
| Thiamine biosynthesis (tyrosine) | M00127 |
| Thiamine biosynthesis (glycine) | M00895 |
| NADH:quinone oxidoreductase | M00144 |
| F-type ATP synthase | M00157 |
| Lysine biosynthesis (succinyl-DAP) | M00016 |
| Lysine biosynthesis (acetyl-DAP) | M00525 |
| Lysine biosynthesis (DAP dehydrogenase) | M00526 |
| Lysine biosynthesis (DAP aminotransferase) | M00527 |
| Pimeloyl-ACP biosynthesis | M00572 |
| Molybdenum cofactor biosynthesis | M00880 |
| Cobalamin biosynthesis (anaerobic) | M00924 |
| Cobalamin biosynthesis (aerobic) | M00925 |

**Supplementary Table III.** Locus tags of proteins for which data is shown in **Figure 3**, **Figure 4** and **Supplementary Figure I**.

| Protein ID | Name in Figure | Locus tag |
| --- | --- | --- |
| QZT34165.1 | ATP synthase ε | HUR95_01715 |
| QZT34166.1 | ATP synthase β | HUR95_01720 |
| QZT34167.1 | ATP synthase γ | HUR95_01725 |
| QZT34168.1 | ATP synthase α | HUR95_01730 |
| QZT34169.1 | ATP synthase δ | HUR95_01735 |
| QZT34170.1 | ATP synthase B | HUR95_01740 |
| QZT34172.1 | ATP synthase A | HUR95_01750 |
| QZT34154.1 | Ndh-1 subunit N | HUR95_01655 |
| QZT34155.1 | Ndh-1 subunit M | HUR95_01660 |
| QZT34156.1 | Ndh-1 subunit L | HUR95_01665 |
| QZT34159.1 | Ndh-1 subunit I | HUR95_01680 |
| QZT34160.1 | Ndh-1 subunit H | HUR95_01685 |
| QZT34161.1 | Ndh-1 subunit D | HUR95_01690 |
| QZT34162.1 | Ndh-1 subunit C | HUR95_01695 |
| QZT34163.1 | Ndh-1 subunit B | HUR95_01700 |
| QZT34164.1 | Ndh-1 subunit A | HUR95_01705 |
| QZT33635.1 | Ndh-2 | HUR95_15605 |
| QZT33594.1 | SdhB | HUR95_15375 |
| QZT33595.1 | SdhA | HUR95_15380 |
| QZT32729.1 | Cyt b6c1 FeS cluster | HUR95_10060 |
| QZT32730.1 | Cyt b6c1 cytochrome b6 | HUR95_10065 |
| QZT32948.1 | Cyt aa-3 subunit I | HUR95_11455 |
| QZT32949.1 | Cyt aa-3 subunit II | HUR95_11460 |
| QZT33452.1 | Cyt ba-3 subunit I | HUR95_14550 |
| QZT33422.1 | Mrp subunit A | HUR95_14385 |
| QZT33805.1 | Iron transporter | HUR95_16600 |
| QZT35027.1 | Cop subunit A | HUR95_07300 |
| QZT33612.1 | Sulfate substrate binding | HUR95_15470 |
| QZT34641.1 | Sao subunit C | HUR95_04675 |
| QZT34643.1 | Sao subunit X | HUR95_04685 |
| QZT33744.1 | Mnt subunit B | HUR95_16235 |
| QZT33746.1 | Mnt subunit D | HUR95_16245 |
| QZT35147.1 | Trk subunit A | HUR95_08000 |
| QZT33333.1 | Trk subunit A | HUR95_13795 |
| QZT34989.1 | Phn subunit D | HUR95_07070 |
| QZT34990.1 | Phn subunit C | HUR95_07080 |
| QZT33306.1 | Phn subunit C | HUR95_13595 |
| QZT35462.1 | Phn substrate binding | HUR95_13600 |
| QZT33012.1 | Flagellum subunit Y | HUR95_11810 |
| QZT33014.1 | Flagellum subunit D | HUR95_11820 |
| QZT33021.1 | Flagellum subunit G | HUR95_11860 |
| QZT32678.1 | Mce | HUR95_09775 |
| QZT32677.1 | Mce | HUR95_09770 |
| QZT35387.1 | MmdA | HUR95_09780 |
| QZT34097.1 | Msdh | HUR95_01280 |
| QZT34941.1 | AccD | HUR95_06775 |
| QZT35340.1 | AccA | HUR95_06780 |
| QZT35382.1 | AccB | HUR95_09605 |
| QZT32651.1 | AccC | HUR95_09610 |
| QZT35103.1 | Pta | HUR95_07730 |
| QZT35102.1 | Ack | HUR95_07725 |
| QZT34540.1 | Acs | HUR95_03960 |
| QZT34238.1 | Acs | HUR95_02150 |
| QZT33266.1 | AcsA | HUR95_13350 |
| QZT34201.1 | Acat | HUR95_01925 |
| QZT34176.1 | Acat | HUR95_01770 |
| QZT34200.1 | Hbd | HUR95_01920 |
| QZT33841.1 | Hadh | HUR95_16815 |
| QZT35250.1 | Bhbd | HUR95_01910 |
